# Predictors of Mortality and Cardiovascular Outcome at 6 Months after Hospitalization for COVID-19

**DOI:** 10.3390/jcm11030729

**Published:** 2022-01-29

**Authors:** Giulia Renda, Fabrizio Ricci, Enrico Guido Spinoni, Leonardo Grisafi, Damiano D’Ardes, Marco Mennuni, Claudio Tana, Andrea Rognoni, Mattia Bellan, Pier Paolo Sainaghi, Mario Pirisi, Simona De Vecchi, Sabina Gallina, Sante Donato Pierdomenico, Francesco Cipollone, Giuseppe Patti

**Affiliations:** 1 Department of Neuroscience, Imaging and Clinical Sciences, G. D’Annunzio University of Chieti-Pescara, 66100 Chieti, Italy; giulia.renda@unich.it (G.R.); fabrizio.ricci@unich.it (F.R.); sabina.gallina@unich.it (S.G.); 2 SS. Annunziata Hospital of Chieti, 66100 Chieti, Italy; damianomatrix89@msn.com (D.D.); claudio.tana@gmail.com (C.T.); sante.pierdomenico@unich.it (S.D.P.); francesco.cipollone@unich.it (F.C.); 3 Department of Clinical Sciences, Lund University, 203 13 Malmö, Sweden; 4 Maggiore della Carità Hospital, 28100 Novara, Italy; enrico.spinoni@gmail.com (E.G.S.); leonardo.grisafi@gmail.com (L.G.); marco.mennuni@gmail.com (M.M.); arognoni@hotmail.com (A.R.); mattia.bellan@med.uniupo.it (M.B.); pierpaolo.sainaghi@med.uniupo.it (P.P.S.); mario.pirisi@uniupo.it (M.P.); simonadevecchi04@gmail.com (S.D.V.); 5 Department of Translational Medicine, University of Eastern Piedmont, 28100 Novara, Italy; 6 Department of Medicine and Science of Aging, G. D’Annunzio University of Chieti-Pescara, 66100 Chieti, Italy; 7 Department of Innovative Technologies in Medicine & Dentistry, G. D’Annunzio University of Chieti-Pescara, 66100 Chieti, Italy

**Keywords:** COVID-19, follow-up, mortality, cardiovascular events

## Abstract

Clinical outcome data of patients discharged after Coronavirus disease 2019 (COVID-19) are limited and no study has evaluated predictors of cardiovascular prognosis in this setting. Our aim was to assess short-term mortality and cardiovascular outcome after hospitalization for COVID-19. A prospective cohort of 296 consecutive patients discharged after COVID-19 from two Italian institutions during the first wave of the pandemic and followed up to 6 months was included. The primary endpoint was all-cause mortality. The co-primary endpoint was the incidence of the composite outcome of major adverse cardiac and cerebrovascular events (MACCE: cardiovascular death, myocardial infarction, stroke, pulmonary embolism, acute heart failure, or hospitalization for cardiovascular causes). The mean follow-up duration was 6 ± 2 months. The incidence of all-cause death was 4.7%. At multivariate analysis, age was the only independent predictor of mortality (aHR 1.08, 95% CI 1.01–1.16). MACCE occurred in 7.2% of patients. After adjustment, female sex (aHR 2.6, 95% CI 1.05–6.52), in-hospital acute heart failure during index hospitalization (aHR 3.45, 95% CI 1.19–10), and prevalent atrial fibrillation (aHR 3.05, 95% CI 1.13–8.24) significantly predicted the incident risk of MACCE. These findings may help to identify patients for whom a closer and more accurate surveillance after discharge for COVID-19 should be considered.

## 1. Introduction

Severe acute respiratory syndrome coronavirus 2 (SARS-CoV-2) is a highly transmissible and pathogenic beta-coronavirus responsible for the pandemic ‘coronavirus disease 2019’ (COVID-19) [1]. Here, most of the available evidence is focused on patients’ characteristics, risk factors, clinical course, and outcome in the acute phase of the infection, particularly among hospitalized cohorts [2,3,4,5,6,7,8,9]. Patients with COVID-19 usually present a respiratory syndrome, including interstitial pneumonia and acute respiratory distress syndrome. However, common complications are a prothrombotic coagulopathy, resulting in venous and arterial thromboembolic events, as well as acute liver or kidney injury and heart involvement characterized by myocarditis, acute coronary events, heart failure, and/or dysrhythmias [8]. To date, follow-up data of patients discharged after COVID-19 are limited [10,11,12,13,14,15] and, in particular, no study has specifically evaluated independent predictors of cardiovascular prognosis in this setting. Hence, the aim of this study was to prospectively assess 6-month mortality and cardiovascular outcome in a multicenter cohort of patients discharged after COVID-19 during the first wave of the pandemic in Italy.

## 2. Methods

Out of 549 patients admitted for COVID-19 in two Italian institutions—Maggiore della Carità Hospital, Novara and Santissima Annunziata Hospital, Chieti—from 20 February through 12 May 2020, we investigated clinical outcome during follow-up among 296 consecutive discharged patients (aged ≥18 years), representing 80% of those discharged alive. SARS-CoV-2 infection was confirmed by reverse-transcriptase-polymerase-chain-reaction assay in all patients. Individual in-hospital data, including demographics, previous medical history, co-morbidities, laboratory results, drug treatments, and clinical outcome, were collected. Patients were enrolled regardless of the type of COVID-19 clinical presentation and in-hospital therapies for the SARS-CoV-2 infection. After discharge, patients were prospectively followed up to 6 months. Follow-up assessment was performed by telephone interviews or ambulatory visits/in-hospital evaluation in the case of clinical recurrence.

The primary endpoint was all-cause mortality at 6 months. The co-primary endpoint was the incidence of the composite outcome measure including major adverse cardiac and cerebrovascular events (MACCE: cardiovascular death, myocardial infarction, stroke, pulmonary embolism, acute heart failure, or hospitalization for cardiovascular causes) at 6 months. The study protocol was approved by the institutional ethical committee (IRB code CE 97/20) and was conducted in strict accordance with the principles of the Declaration of Helsinki.

### Statistical Analysis

The normality of distribution of the parameters was assessed by Kolmogorov–Smirnov test. Since all continuous variables had a normal distribution, they were described as mean ± standard deviation. Categorical variables were expressed as frequencies and percentages. One-way ANOVA test was used for group differences in continuous variables and Fisher exact test for group differences in categorical variables. The follow-up time was estimated as the time between hospital discharge and date of event or end of follow-up through 31 December 2020. Kaplan–Meier analysis for all-cause mortality and MACCE was performed. The Schoenfeld residuals test was used to check the proportional hazards assumption. Cox regression model was applied to estimate hazard ratios with a 95% confidence interval (CI). The Cox regression multivariable model was adjusted for age, sex, and those variables showing an association with *p* < 0.10 at the univariate model. There were no missing values in any of the outcomes. All calculations were performed using the Wizard 2 statistical software version 2.0.4 for Mac and Prism 9 (1995–2022 GraphPad Software, LLC, La Jolla, CA, USA). All tests were two-sided and a *p* value < 0.05 was considered statistically significant.

## 3. Results

The main characteristics of the study population at baseline (n = 296) are reported in Table 1. The mean age was 64 ± 16 years, and the prevalence of male sex was 58%. Women were more frequently smokers (*p* = 0.021), more frequently affected by chronic kidney disease (*p* = 0.032), atrial fibrillation (*p* = 0.01), and cognitive impairment (*p* = 0.003) compared with men. 

**Figure 1:**
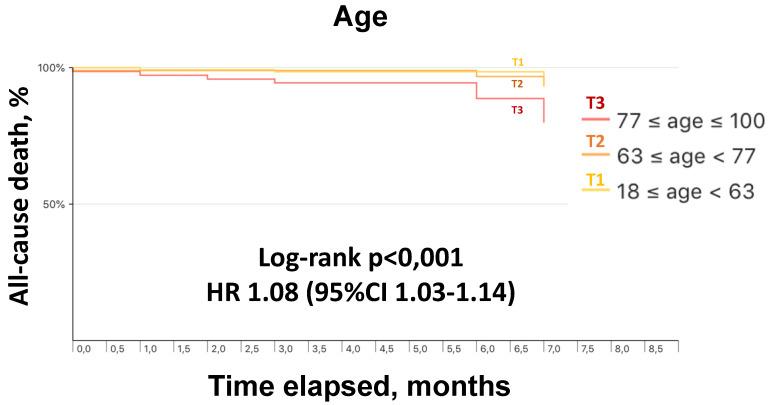
Kaplan–Meier survival curves at 6 months by tertiles of age.

The mean follow-up duration was 6 ± 2 months. Follow-up data were obtained in all patients. The incidence of all-cause death during follow-up was 4.7% (14 events) (Table 2). As compared with survivors, deceased patients were significantly older; had a higher prevalence of peripheral artery disease and atrial fibrillation; during the index hospitalization, suffered more frequently from acute heart failure and ischemic stroke, and showed higher neutrophil-to-lymphocyte ratio and lower estimated glomerular filtration rate; had increased prevalence of left bundle branch block; presented a greater use of beta-blockers, diuretic agents, and oral anticoagulant therapy (Table 1). Length of stay during index hospitalization was similar in survivors and deceased patients (13 ± 9 vs. 14 ± 10 days; *p* = 0.61). At univariate analysis, age, in-hospital acute heart failure, QRS duration at baseline electrocardiogram, and in-hospital use of beta-blockers were associated with higher mortality during follow-up (Table 3). After adjustment, age remained the only independent predictor of all-cause death (aHR 1.08; 95% CI 1.01–1.16) (Table 3). Figure 1 shows survival curves at 6 months according to tertiles of age.

**Figure 2:**
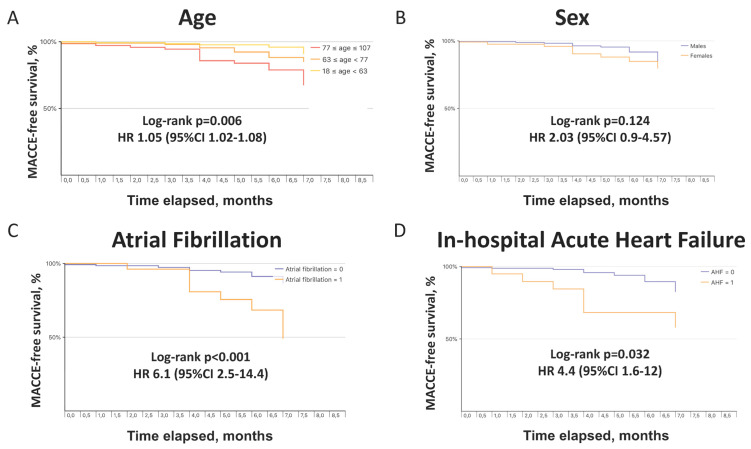
Kaplan–Meier curves showing MACCE-free survival at 6 months by tertiles of age (**A**), sex (**B**), atrial fibrillation status (**C**), and occurrence of acute heart failure during index hospitalization for COVID-19 (**D**). COVID-19, coronavirus disease 2019; MACCE, major adverse cardiac and cerebrovascular events.

MACCE after discharge occurred in 21 patients (7.2%). Crude rates of individual adverse events included in the composite cardiovascular outcome are reported in Table 2. As compared with those without events, patients with MACCE were significantly older; had a higher prevalence of peripheral artery disease, atrial fibrillation, chronic obstructive pulmonary disease, and chronic kidney disease; during the index hospitalization, suffered more frequently from deep venous thrombosis and showed higher neutrophil-to-lymphocyte ratio and PaCO_2_; presented a greater use of beta-blockers, diuretic agents, and anticoagulant therapy (Table 1). At multivariate analysis, female sex (aHR 2.6, 95% CI 1.05–6.52), prevalent atrial fibrillation (aHR 3.05, 95% CI 1.13–8.24), and in-hospital acute heart failure (aHR 3.45, 95% CI 1.19–10) were independent predictors of MACCE (Table 3). MACCE-free survival curves at 6 months according to tertiles of age, sex, prevalent atrial fibrillation, and in-hospital acute heart failure are depicted in Figure 2.

## 4. Discussion

In this prospective, multicenter investigation we first provided 6-month follow-up data on mortality and cardiovascular morbidity among patients discharged after COVID-19 during the first wave of the current pandemic in Italy. We observed a mortality rate of 4.7% and a crude MACCE incidence of 7.2%. Age resulted as the sole independent predictor of all-cause death, whereas female sex, in-hospital acute heart failure, and prevalent atrial fibrillation were independent predictors of MACCE.

Evidence on clinical outcomes during follow-up of patients discharged for COVID-19 is scant. Two studies explored the persistence of symptoms at 2 months, showing at least one symptom, particularly fatigue and dyspnea, in 87% of patients with more severe COVID-19, and 68% of those with a non-critical disease, mainly anosmia/ageusia, dyspnea, or asthenia [12,13]. Other investigations reported a high incidence of residual impairment of pulmonary function and lung injury by computed tomography performed at 3 months after discharge in survivors of critical COVID-19 [14,15]. Furthermore, data on residual physical and functional impairment at 3 to 6-month follow-up [10], as well as on the persistence of psychological sequelae at 4 months [11], have been recently published. Readmission and death rate at 60 days was evaluated in the nationwide Veterans Affairs health care system, without analyzing organ-specific endpoints [16]. The largest study evaluating organ-specific dysfunction in individuals with COVID-19 after discharge included 47,780 English patients over a follow-up of 140 days, and observed an increased risk of mortality, readmission, and multiorgan dysfunction compared with similar individuals in the general population [17]. More recently, in a German cohort of patients hospitalized for COVID-19, 6-month all-cause mortality and readmission rates were related to coagulopathy, congestive heart failure, neurological diseases, and acute renal failure, while the female sex resulted in a protective factor [18].

The present study represents the first report specifically focused on independent predictors of mortality and cardiovascular outcome in survivors after COVID-19 hospitalization.

In 2019 the probability of annual death for individuals aged 64 years (i.e., the mean age in our study population) in Italy was 0.7% [19]. In patients discharged after COVID-19, we observed a crude mortality > 6 times higher over 6 months, with a cardiovascular event being the cause of death in 43% of patients. Importantly, in our investigation overall mortality after the hospitalization was unrelated to severity of COVID-19-related respiratory impairment at presentation, length of stay, or occurrence of in-hospital complications, also including the need for intensive care unit admission. Older age has been shown an independent predictor of lower in-hospital survival in patients with COVID-19 [9]. In particular, described in-hospital mortality overall ranges between 15% and 20%, but varies across decades of age and exceeds 60% in octogenarians [6]. This reflects frailty, prevalent co-morbidities, and higher rates of complications with aging. The present study indicates over 6 months an 8% age-related overall relative increase in all-cause death and a 10% absolute increase in mortality in the subgroup of patients with age in the highest tertile (>77 years).

Prevalent cardiovascular diseases are frequent in patients hospitalized for COVID-19 [6], but little is known about their incidence and prognostic significance after discharge. We demonstrated a not negligible overall incidence of cardiovascular events at 6-month follow-up. Atrial fibrillation is a common feature in patients hospitalized for COVID-19, partly because it shares with such disease a high prevalence of older age, cardiovascular risk factors, and co-morbidities, and partly because it represents a frequent new-onset complication. In these patients, atrial fibrillation has been reported in approximately 20% of cases (either historical or new-onset) [20], and such arrhythmia, especially new-onset, resulted in an independent predictor of in-hospital all-cause death, cardiovascular death, and more severe clinical pattern [21]. It has been hypothesized that SARS-CoV-2 infection-related inflammation, edema, and fibrosis of atrial tissue, besides immune response, hypoxia, and electrolyte abnormalities, can contribute to the occurrence of atrial arrhythmias, in particular atrial fibrillation [20,22]. Notably, at multivariate analysis, we found that atrial fibrillation was associated with a three-fold increase of cardiovascular events at 6 months after discharge. Atrial fibrillation as a marker of increased cardiovascular risk, as well as a more severe cardiac impairment in patients with atrial fibrillation, may explain the excess in mortality related to this arrhythmia, either during the in-hospital stay or afterward during follow-up.

We also observed that an acute heart failure event during index hospitalization was independently associated with a 3.5-fold higher risk of MACCE after discharge. This may reflect an underlying cardiac impairment persisting over time and predisposing to further adverse events during follow-up. Unfortunately, we had no data on the specific causes of acute heart decompensation during the in-hospital stay and we cannot discern whether it occurred in patients with pre-existing cardiac diseases or was precipitated by new cardiovascular events, either spontaneous or related to SARS-CoV-2 infection, such as acute coronary syndromes, myocarditis, arrhythmias, respiratory failure, renal insufficiency, sepsis. A possible explanation for cardiovascular events occurring during the months after discharge is that inflammation and immune reaction persist for a longer period relative to hospitalization and continue to affect the cardiovascular system. On the other hand, clinical features and co-morbidities of COVID-19 patients may account for the increased cardiovascular risk.

Moreover, in our study female sex was an independent predictor of MACCE. This appears to be in contrast with a reported higher incidence of complications and mortality among male patients during the acute phase of SARS-CoV-2 infection [5,6,7,8,9]. Sex differences in both innate and adaptative immune systems, related to hormones and cytokines production, have been hypothesized to explain such survival advantage in women [23]. Indeed, a previous investigation found that the female sex was associated with a higher risk of respiratory sequelae at 4 months after discharge for COVID-19 [11]. To date, only one study showed a lower rate of all-cause death at 6-month follow-up in women compared to men [18] and no data are available on possible sex-related differences in terms of cardiovascular prognosis during follow-up in patients with COVID-19. We observed a 2.6-fold increased risk of MACCE at 6 months in female vs male patients that might likely be explained by an unbalanced distribution of frailty-related conditions, including chronic kidney disease, atrial fibrillation, and cognitive impairment, more frequently observed in women. Furthermore, the largely reported excess in-hospital mortality in men [5,6,7,8,9] could justify a relatively greater number of women at risk of suffering adverse events after COVID-19 hospitalization.

Our study has strengths and limitations. Strengths include the robustness of data obtained from a multicenter, real-life population with a wide spectrum of COVID-19-related clinical features, also including a severe pulmonary disease; the reliability of prospectively collected data with a comprehensive assessment of individual medical history, medical treatments, in-hospital outcome, and follow-up evaluation. Limitations include the risk of inclusion bias, despite the study aiming to enroll consecutive patients; residual confounding, due to the lack of adjustment for all potential confounders; the absence of information on B-type natriuretic peptides, d-dimer levels, and echocardiographic features at the time of discharge, as well as on specific causes of non-cardiovascular death at 6 months; and the follow-up assessment being performed by telephone interviews in a large proportion of patients. However, the latter was indispensable due to rigorous access restrictions in the hospital for all patients requiring elective cardiological visits during the COVID-19 pandemic in Italy.

In conclusion, this prospective, multicenter investigation first addresses the issue of cardiovascular outcome at 6 months in patients hospitalized for COVID-19. Our findings may help to detect patients at higher risk of adverse events after discharge for whom a closer and more accurate clinical and imaging surveillance should be considered.

## Figures and Tables

**Table 1:** Demographic and clinical characteristics in the study population by event status.

	Overall (n = 296)	Deceased during Follow-Up (n = 14)	Survivors (n = 282)	*p* Value	MACCE during Follow-Up (n = 21)	No MACCE (n = 275)	*p* Value
**Baseline characteristics**							
Age—year, mean ± SD	64 ± 16	77 ± 14	64 ± 16	**0.002**	75 ± 17	63 ± 16	**0.002**
Male sex, n (%)	172 (58)	7 (50)	165 (59)	0.585	8 (38)	161 (59)	0.107
Caucasian, n (%)	246 (93)	12 (86)	234 (83)	0.568	18 (86)	224 (81)	0.776
Weight—kg, mean ± SD	76 ± 14	68 ± 12	77 ± 14	0.126	72 ± 10	77 ± 14	0.219
BMI, mean ± SD	27 ± 4	24 ± 3	27 ± 4	0.244	27 ± 4	27 ± 4	0.999
Arterial hypertension, n (%)	149 (50)	9 (64)	140 (50)	0.413	10 (48)	137 (50)	0.999
Dyslipidemia, n (%)	59 (20)	1(7)	58 (21)	0.316	6 (29)	53 (19)	0.392
Diabetes mellitus, n (%)	53 (18)	4 (29)	49 (17)	0.287	3 (14)	51 (18)	0.776
Current smoking, n (%)	25 (8)	2 (14)	23 (8)	0.335	4 (19)	25 (9)	0.137
Cardiomyopathy, n (%)	51 (17)	5 (36)	46 (16)	0.073	6 (29)	45 (16)	0.224
Previous PCI, n (%)	24 (8)	1 (7)	23 (8)	0.999	3 (14)	22 (8)	0.402
Previous CABG, n (%)	4 (1)	0 (0)	4 (1)	0.823	0 (0)	5 (2)	0.999
AF, n (%)	18 (6)	3 (21)	15 (5)	**0.045**	4 (19)	14 (5)	**0.030**
PAD, n (%)	30 (10)	4 (29)	26 (9)	**0.042**	6 (28)	27 (10)	**0.019**
COPD, n (%)	24 (8)	0 (0)	24 (8)	0.613	5 (24)	25 (9)	**0.048**
OSAS, n (%)	5 (2)	0 (0)	5 (2)	0.999	0 (0)	6 (2)	0.999
ILD during index hospitalization, n (%)	5 (2)	0 (0)	5 (2)	0.999	1 (5)	5 (2)	0.359
CKD, n (%)	32 (11)	3 (21)	29 (10)	0.183	8 (38)	31 (11)	**0.002**
History of cancer, n (%)	43 (14)	6 (43)	37 (13)	0.183	5 (24)	38 (14)	0.205
Chronic liver disease, n (%)	7 (2)	1 (7)	6 (2)	0.290	1 (5)	9 (3)	0.527
Autoimmune disease, n (%)	11 (4)	0 (0)	11 (4)	0.999	1 (5)	13 (5)	0.999
Pevious organ transplant, n (%)	4 (1)	0 (0)	4 (1)	0.999	0 (0)	5 (2)	0.999
Cognitive impairment, n (%)	29 (10)	3 (21)	26 (9)	0.147	2 (10)	33 (12)	0.999
**Signs upon admission for COVID-19**							
Temperature—°C, mean ± SD	37.5 ± 1.1	37.2 ± 1.2	37.5 ± 1.1	0.301	37.0 ± 1.0	37.5 ± 1.0	0.037
Systolic blood pressure—mmHg, mean ± SD	127 ± 20	129 ± 26	127 ± 20	0.730	129 ± 29	127 ± 19	0.517
Diastolic blood pressure—mmHg, mean ± SD	74 ± 11	74 ± 9	74 ± 12	0.928	72 ± 13	73 ± 12	0.720
Heart Rate—bpm, mean ± SD	85 ± 16	83 ± 21	86 ± 16	0.466	81 ± 17	87 ± 16	0.133
Respiratory rate—bpm, mean ± SD	20 ± 5	19 ± 3	21 ± 5	0.241	21 ± 4	20 ± 5	0.862
Oxygen saturation—%, mean ± SD	95 ± 4	94 ± 4	95 ± 4	0.526	93 ± 6	92 ± 6	0.644
**Laboratory data upon admission for COVID-19**							
WBC—n/mm^3^, mean ± SD	7091 ± 3371	7723 ± 3772	7057 ± 3353	0.489	7320 ± 2592	7118 ± 3455	0.799
Neutrophil—%, mean ± SD	70 ± 12	72 ± 11	70 ± 12	0.620	69 ± 12	71 ± 11	0.544
Lymphocites—%, mean ± SD	21 ± 10	17 ± 11	21 ± 9	0.205	19 ± 9	20 ± 10	0.638
NLR, mean ± SD	4.5 ± 5.6	10.7 ± 18.9	4.1 ± 3.6	**<0.001**	7.8 ± 16	4 ± 4	**0.017**
Hemoglobin—g/dL, mean ± SD	13.4 ± 1.7	13.0 ± 1.0	13.4 ± 1.7	0.490	13 ± 2	13 ± 2	0.162
Platelets—n/mm^3^, mean ± SD	211 ± 76	213 ± 79	211 ± 77	0.888	219 ± 72	212 ± 79	0.695
CRP—mg/L, mean ± SD	21 ± 42	21 ± 33	21 ± 42	0.972	28 ± 39	23 ± 45	0.657
Creatinine—mg/dL, mean ± SD	0.9 ± 0.6	1.0 ± 0.4	0.9 ± 0.6	0.588	1.4 ± 2.2	1 ± 0.7	0.056
eGFR—mL/min, mean ± SD	81 ± 28	58 ± 24	82 ± 27	**0.025**	67 ± 27	80 ± 29	0.059
**ABG upon admission for COVID-19**							
pH, mean ± SD	7.45 ± 0.06	7.46 ± 0.047	7.46 ± 0.06	0.663	7.44 ± 0.07	7.40 ± 0.64	0.754
PaCO2—mmHg, mean ± SD	35 ± 6	35 ± 4	35 ± 7	0.920	38 ± 13	35 ± 7	**0.045**
PaO2—mmHg, mean ± SD	68 ± 19	66 ± 17	68 ± 19	0.812	72 ± 19	67 ± 18	0.271
HCO3—mEq/L, mean ± SD	25 ± 4	26 ± 4	25 ± 4	0.945	27 ± 8	25 ± 4	0.137
SaO2—%, mean ± SD	91 ± 11	92 ± 7	91 ± 11	0.960	93 ± 6	91 ± 11	0.575
Lactate—mmol/L, mean ± SD	1.2 ± 0.9	1.2 ± 0.5	1.2 ± 0.9	0.918	1.5 ± 1.1	1.2 ± 0.9	0.189
PaO2/FiO2 ratio, mean ± SD	299 ± 82	300 ± 62	299 ± 83	0.975	332 ± 92	294 ± 83	0.050
**ECG upon admission for COVID-19**							
QRS duration—ms, mean ± SD	25 ± 22	118 ± 45	94 ± 20	**<0.001**	97 ± 20	95 ± 20	0.648
QTc interval—ms, mean ± SD	436 ± 32	443 ± 51	436 ± 32	0.409	445 ± 27	436 ± 32	0.214
LBBB, n (%)	18 (6)	4 (28)	14 (5)	**0.007**	2 (10)	17 (6)	0.634
RBBB, n (%)	15 (5)	0 (0)	15 (5)	0.999	1 (5)	14 (5)	0.999
**Therapy**							
Beta-blockers, n (%)	82 (28)	10 (71)	72 (26)	**<0.001**	10 (48)	69 (25)	**0.038**
CCBs, n (%)	59 (20)	2 (14)	57 (20)	0.744	5 (24)	53 (19)	0.575
Oral diuretic drugs, n (%)	68 (23)	7 (50)	61 (22)	**0.008**	9 (43)	56 (20)	**0.026**
Intravenous diuretic drugs, n (%)	24 (8)	4 (29)	20 (7)	**0.019**	3 (14)	19 (2)	0.197
Nitrates, n (%)	9 (3)	2 (14)	7 (2)	0.062	0 (0)	6 (2)	0.999
Anti-arrhythmics, n (%)	17 (6)	1 (7)	16 (6)	0.571	0 (0)	9 (3)	0.999
ASA, n (%)	51 (17)	2 (14)	49 (17)	0.999	4 (19)	51 (18)	0.999
P2Y12 inhibitors, n (%)	9 (3)	0 (0)	9 (3)	0.999	1 (5)	12 (4)	0.999
OAC, n (%)	17 (6)	3 (21)	14 (5)	**0.039**	7 (33)	15 (6)	**<0.001**
ACE-inhibitors, n (%)	43 (15)	2 (14)	41 (15)	0.999	2 (10)	48 (17)	0.546
ARBs, n (%)	22 (7)	1 (7)	21 (7)	0.999	1 (5)	31 (11)	0.712
Insulin, n (%)	36 (12)	1 (7)	35 (12)	0.999	3 (14)	11 (4)	0.067
Statins, n (%)	24 (8)	0 (0)	24 (9)	0.613	3 (14)	46 (17)	0.999
Oral antidiabetic drugs, n (%)	8 (3)	0 (0)	8 (3)	0.999	1 (5)	24 (9)	0.999
QTc modifying drugs, n (%)	166 (56)	10 (71)	156 (55)	0.280	1 (5)	40 (15)	0.328
Hydroxycloroquine, n (%)	240 (81)	9 (64)	231 (82)	0.152	14 (67)	223 (81)	0.151
Lopinavir, n (%)	47 (16)	1 (7)	46 (16)	0.705	3 (14)	43 (14)	0.999
Remdesivir, n (%)	4 (1)	0 (0)	4 (1)	0.999	0 (0)	4 (1)	0.999
Darunavir, n (%)	115 (39)	4 (29)	111 (39)	0.576	5 (24)	109 (40)	0.170
Tocilizumab, n (%)	8 (3)	0 (0)	8 (3)	0.999	0 (0)	8 (3)	0.999
LMWH, n (%)	202 (68)	9 (64)	193 (68)	0.772	17 (81)	182 (66)	0.228
Azithromycine, n (%)	64 (22)	3 (21)	61 (22)	0.999	3 (14)	61 (22)	0.583
Steroids, n (%)	46 (16)	0 (0)	46 (16)	0.137	4 (19)	42 (15)	0.548
**In-hospital events**							
Acute heart failure, n (%)	20 (7)	4 (29)	16 (6)	**0.001**	4 (19)	20 (7)	0.078
ALI, n (%)	103 (35)	3 (21)	100 (35)	0.393	11 (52)	101 (37)	0.167
ARDS, n (%)	50 (17)	1 (7)	49 (17)	0.477	3 (14)	53 (219)	0.774
AKI, n (%)	13 (4)	1 (7)	12 (4)	0.474	0 (0)	20 (7)	0.379
CRRT, n (%)	6 (2)	0 (0)	6 (2)	0.999	1 (5)	11 (4)	0.593
Secondary infection, n (%)	35 (12)	2 (14)	33 (12)	0.674	2 (10)	42 (15)	0.750
Septic shock, n (%)	1 (0)	0 (0)	1 (0)	0.999	1 (5)	3 (1)	0.256
Any thrombotic complication, n (%)	13 (4)	1 (7)	12 (4)	0.474	2 (10)	14 (5)	0.316
ACS, n (%)	2 (1)	0 (0)	2 (1)	0.999	0 (0)	5 (2)	0.999
Pulmonary embolism, n (%)	8 (3)	0 (0)	8 (3)	0.999	1 (5)	7 (3)	0.449
Deep venous thrombosis, n (%)	4 (1)	0 (0)	4 (1)	0.999	2 (10)	3 (1)	**0.042**
Ischemic stroke, n (%)	1 (0)	1 (7)	0 (0)	**0.047**	0 (0)	1 (0.4)	0.999
Bilateral CT involvment, n (%)	168 (57)	6 (43)	162 (57)	0.408	12 (57)	167 (61)	0.818
ICU admission, n (%)	24 (8)	1 (7)	23 (8)	0.999	0 (0)	23 (8)	0.388
In-hospital LOS—days, mean ± SD	14 ± 10	13 ± 9	14 ± 10	0.612	16 ± 9	13 ± 10	0.355

ABG: arterial blood gas analysis; ACE: angiotensin converting enzyme; ACS: acute coronary syndromes; AF: atrial fibrillation; AKI: acute kidney injury; ALI: acute lung injury; ARBs: angiotensin receptor blockers; ASA: acetylsalicylic acid; BMI: body mass index; CABG: coronary artery bypass graft; CCBs: calcium channel blockers; CKD: chronic kidney disease; COPD: chronic obstructive pulmonary disease; COVID-19: Coronavirus disease 2019; CRP: C reactive protein; CRRT: continuous renal replacement therapy; CT: computed tomography; eGFR: estimated glomerular filtration rate; ICU: intensive care unit; ILD: interstitial lung disease; LBBB: left bundle branch block; LMWH: low molecular weight heparin; LOS: length of stay; MACCE: major adverse cardiac and cerebrovascular events; NLR: neutrophil-lymphocyte ratio; OAC: oral anticoagulant therapy; OSAS: obstructive sleep apnea syndrome; PAD: peripheral artery disease; PCI: percutaneous coronary intervention; RBBB: right bundle branch block; WBC: white blood cells. Significant *p* values are reported in **bold.**

**Table 2:** Six-month crude event rates in patients discharged after COVID-19.

Outcome	Number of Events	Crude Event Rate (%)	95% CI
All-cause death	14	4.730	2.252–7.207
MACCE	21	7.095	4.060–10.129
Cardiovascular death	6	2.027	0.405–3.649
Myocardial infarction	2	0.676	0.000–1.612
Stroke	4	1.351	0.027–2.676
Pulmonary embolism	1	0.338	0.000–1.000
Acute heart failure	6	2.027	0.405–3.649
Hospitalization for cardiovascular causes	14	4.730	2.252–7.207

CI: confidence interval; COVID-19: Coronavirus disease 2019; MACCE: major adverse cardiac and cerebrovascular events.

**Table 3:** Univariate and multivariate Cox regression analysis.

All-Cause Death								
Covariate	Univariate				Multivariate			
	HR	95% CI	Z-Score	*p* Value	HR	95% CI	Z-Score	*p* Value
**Age**	1.083	1.03–1.139	3.106	0.002	1.083	1.008–1.165	2.163	**0.031**
**Female sex**	1.507	0.475–4.781	0.696	0.487	1.683	0.393–7.198	0.702	0.483
**In-hospital acute heart failure**	5.414	1.399–20.948	2.447	0.014	2.003	0.437–9.193	0.894	0.371
**QRS duration**	1.031	1.011–1.052	3.031	0.002	1.015	0.999–1.042	1.183	0.237
**In-hospital beta-blockers use**	8.489	2.174–33.152	3.077	0.002	1.887	0.397–8.97	0.799	0.424
**MACCE**								
**Covariate**	**Univariate**				**Multivariate**			
	**HR**	**95% CI**	**Z-Score**	***p* Value**	**HR**	**95% CI**	**Z-Score**	***p*** **Value**
**Age**	1.049	1.016–1.082	2.985	0.003	1.026	0.99–1.064	1.425	0.154
**Female sex**	2.029	0.9–4.571	1.707	0.088	2.612	1.047–6.518	2.058	**0.040**
**In-hospital acute heart failure**	4.39	1.604–12.012	2.88	0.004	3.454	1.193–9.999	2.286	**0.022**
**AF**	6.077	2.555–14.452	4.082	<0.001	3.049	1.128–8.24	2.198	**0.028**

AF: atrial fibrillation; CI: confidence interval; MACCE: major adverse cardiac and cerebrovascular events. Significant *p* values at multivariate analysis are reported in **bold.**

## Data Availability

The authors agree to make data and materials supporting the results or analyses presented in their paper available upon reasonable request.
